# Anthropogenic Rare Earth Elements: Gadolinium in a Small Catchment in Guizhou Province, Southwest China

**DOI:** 10.3390/ijerph16204052

**Published:** 2019-10-22

**Authors:** Jue Zhang, Zhuhong Wang, Qixin Wu, Yanling An, Huipeng Jia, Yuanyi Shen

**Affiliations:** 1 Key Laboratory of Karst Environment and Geohazard, Ministry of Land and Resources, the College of Resources and Environmental Engineering, Guizhou University, Guiyang 550025, China; juezhang272@163.com (J.Z.); jhp136318@163.com (H.J.); syy136318@163.com (Y.S.); 2 Key Laboratory of Environmental Pollution Monitoring and Disease Control, Ministry of Education, School of Public Health, Guizhou Medical University, Guiyang 550025, China; 3 Guizhou Institute of Technology, Guiyang 550025, China; 20170792@git.edu.cn

**Keywords:** gadolinium, anthropogenic, rare earth elements, dissolved

## Abstract

Rare earth elements (REEs), known as “industrial vitamins”, are widely used in medical treatment, industry, agriculture, etc. However, with the increasing demand for REEs, excess REEs, such as gadolinium (Gd), are considered micropollutants in the environment. In this paper, the distributions of dissolved REEs were analyzed in three small streams, in order to determine the extent and occurrence of Gd anomalies. The shale-normalized REE patterns in the three streams were less smooth with heavy REEs higher than light REEs, for a weak reaction of the heavy REE complexes. A negative Ce (cerium) anomaly and positive samarium (Sm) and europium (Eu) anomalies were observed in the three streams and the negative Ce anomaly was affected by the pH of the alkaline rivers. However, a positive Gd anomaly was found in only a typical urban small stream, Jinzhong. With a population of approximately 60,000, Jinzhong runs by a hospital and through wastewater treatment plants (WWTPs). The concentrations of Gd in Jinzhong ranged from 1.54 to 86.65 ng/L with high anthropogenic Gd proportions (63.64%–98.07%). Anthropogenic Gd showed significant seasonal variations and distinct spatial disparities from upstream to downstream, and it was associated with certain ions such as Cl^−^. Anthropogenic Gd could be attributed to gadopentetic acid (Gd-DTPA), which is used in magnetic resonance imaging (MRI) in hospitals. This type of Gd was shown to be correlated with municipal wastewater. Due to the high stability and low particulate reactivity in water, anthropogenic Gd has great potential to serve as a tracer to prove the presence of medical wastewater.

## 1. Introduction

Consisting of 16 critical elements, rare earth elements (REEs) could be divided into three groups: (1) Light rare earth elements (LREE, from lanthanum to neodymium); (2) Middle rare earth elements (MREE, from praseodymium to holmium); (3) Heavy rare earth elements (HREE, from erbium to lutetium, including yttrium). As good tracers in rivers, REEs in a dissolved load play a significant role in sediments sources [1], groundwater mixing [2], and some geochemical processes in water systems [3,4]. Furthermore, with unique physical and chemical properties, REEs are widely applied in industry and agriculture (e.g., cell phones, glass additives, catalytic converters, fiber optics, rechargeable batteries, electric cars, and fertilizers) [5,6,7]. In recent years, with the explosive demand for REEs, the enrichment of REEs in the environment and their possible health risks to humans have resulted in substantial concerns [8,9,10]. Following anthropogenic gadolinium (Gd), the anomalies of lanthanum (La), samarium (Sm), and europium (Eu) in aquatic systems have been reported [10,11,12,13,14,15]. Notably, anthropogenic REEs have been considered micropollutants in the water [13,16].

During the past few decades, dozens of papers about anthropogenic REE anomalies in aquatic systems have been published. In 1996, anthropogenic Gd anomalies were first found in German rivers [17]. Since that time, positive Gd anomalies have also been reported in some countries, including France [12], Poland [18], Japan [19], and the United States [13,20]. The gadopentetic acid (Gd-DTPA) used in magnetic resonance imaging (MRI) is considered to be the major source of the positive Gd anomalies in water systems [17]. As a paramagnetic contrast agent, Gd-DTPA could be stable under natural conditions for at least six months [21]. The organic complexes of Gd are excreted from human bodies readily into the water system [14,17]. Because of their high stability and low particulate reactivity, they are unlikely to be removed while passing through wastewater treatment plants [21,22]. Therefore, the enrichment of Gd in water systems may have a potential impact on water quality and human health, such as nephrogenic systemic fibrosis, a serious late adverse reaction to Gd-based contrast agents [23].

The research on dissolved REEs in Chinese aquatic systems that has occurred in recent years has mainly concentrated on, (1) the distribution patterns and complex states of dissolved REEs [24,25]; (2) groundwater chemistry research [26,27]; and (3) tracers of sources [28]. Few investigations of Gd enrichment in aquatic systems have been reported in China. Mao et al. [29] observed the REE geochemistry in surface floodplain sediments in the Xiangjiang River in 2014, and this study showed enriched anthropogenic REEs in MREE. No distinct positive Gd anomaly was found.

In the past two decades, the medical community has grown rapidly in China. In Guiyang, the capital of Guizhou, the number of large-scale hospitals increases each year, as occurs in most cities in China. High-tech medical devices, such as MRI, are becoming more common in hospitals, which increases the use of the paramagnetic contrast agent Gd with a likely discharge into the environment.

In this study, we conducted a systematic assessment of REEs in three small streams located in a small catchment in Guizhou, southwestern China. The consistent geological background eliminates interference from geology. Among the three streams, the Jinzhong stream flows through an urban area with a sewage treatment plant and a hospital, while the other two streams are surrounded by agricultural land with low population density. With few studies on Gd anomalies in water systems in China, this study is the first systematic study of anthropogenic Gd that affects the distribution pattern of rare earth elements in rivers. The objectives of this study are to: (1) understand the occurrence of Gd anomalies and their potential as tracers for medical wastewater; and (2) explore the extent of anthropogenic Gd in human-impacted streams.

## 2. Materials and Methods

### 2.1. Study Site

**Figure 1:**
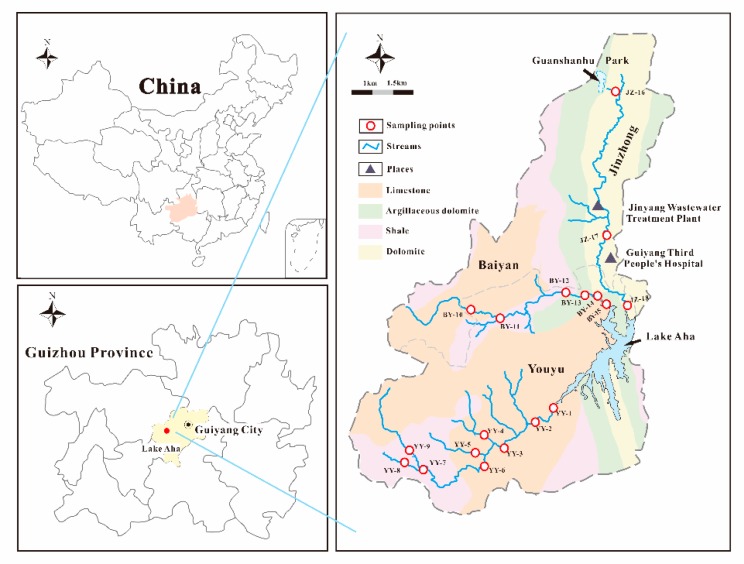
Sampling points of the three tributaries of Lake Aha (YY: Youyu Stream, BY: Baiyan Stream, JZ: Jinzhong Stream).

The Lake Aha basin is located in Guizhou Province, Southwest China (N 26°34′, E 106°43′) [30]. The investigated streams, included the Youyu, Baiyan, and Jinzhong, the three tributaries of the Lake Aha in Guiyang (Figure 1). Permian limestone and Triassic shale are greatly exposed in the Youyu, while Baiyan is Triassic dolomite and shale. Triassic dolomite is distributed in the Jinzhong. The Youyu drains an area of 61.9 km^2^ and has a length of 18.5 km entering the Lake Aha from west. Surrounded by 11 villages, the land types of Youyu are mainly agricultural land, with less residential and commercial land (3.3%). The Baiyan is the main stream of the Lake Aha with a 51.5 km^2^ drain area and 15 km length. Flowing through six villages, Baiyan are less affected by industrial activities. Unlike the other two streams, the Jinzhong runs through a densely populated area, with 60,000, inhabitants. The Jinzhong has a length of 16.5 km, over a drain area of 47.5 km^2^. Originating from the Gaoxin District, the Jinzhong watershed consists mainly of residential and commercial land (81.6%), while agricultural land only accounts for 6.4%. Covered by the urban areas, the Jinzhong is a typical urban small stream impacted by intense human activities.

### 2.2. Sampling and Analytical Procedure

The water used in this research was 18.2 MΩcm Milli-Q water. HNO_3_ was purified before use. All polyethylene bottles were washed with Milli-Q water before using them. From May 2017 to May 2018, surface water samples were collected once every month from 18 sampling points of Youyu (YY), Baiyan (BY) and Jinzhong (JZ) stream (depth below 10 cm). 1–9 sampling points were located in Youyu, and 10–15 sites were located in Baiyan. The 16–18 sites were located in the upstream, downstream and middle reaches of the Jinzhong (Figure 1). A total of 234 surface water samples, including the samples of the rainy season (May to October 2017) and dry season (November 2017 to April 2018), were collected and analyzed.

The pH was measured by the WTW Multi3430 (WTW Company, Weilheim, Upper Bavaria, Germany) at the sampling points. The samples were immediately filtered through cellulose-acetate membrane filters (0.22 μm, Whatman) into the 2 L polyethylene bottles after collection and then acidified (pH < 2) with certain amounts purified HNO_3_ for sample conservation. Then, the Inductively Coupled Plasma Mass Spectrometry (ICP-MS) was used for REE analysis, NexION300X ICP-MS (Perkin Elmer, Waltham, MA, USA) at the Institute of Geochemistry, Chinese Academy of Sciences. Rh was as an internal standard for ICP-MS chemical procedure on REE measurement. The accuracy of the analysis was estimated to be <3% and the analytical quality was checked by SLRS-5 natural water samples from the National Research Council of Canada (NRC). And the anions (SO_4_^2−^, Cl^−^) were analyzed by ion chromatograph (DIONEX, ICS-1100, Sunnyvale, CA, USA).

### 2.3. Data Treatment

To describe the patterns of rare earth elements, the REEs were normalized by the Post-Archean Australia Shale (PAAS) [31]. In this study, we focused on anthropogenic Gd anomaly. According to previous research, there were several methods to calculate REEs anomalies, including extrapolation and interpolation [32,33], or a third order polynomial fit with the PAAS-normalized REE pattern [14,22]. These methods could be used to analyze the extent of REE anomalies and anthropogenic input ratios. Yet there might be errors using interpolation method, especially to the steep normalized REE patterns [14,31]. In 2016, Hatje et al. [14] tested different approaches on Gd anomaly calculation and found that the approach that interpolates europium and neodymium could obtain the smallest anomaly. Other elements employed in the Gd anomaly interpolation (e.g., samarium (Sm) and dysprosium (Dy); neodymium (Nd) and dysprosium (Dy)) and polynomial fit with all REEs except cerium (Ce) and europium (Eu) were also mentioned [14]. Thus the verification of the employed Gd anomaly was crucial. Considering the data for the REEs in our research, the positive Gd anomaly was obtained by interpolation method using neodymium and dysprosium with insignificant difference (*p* < 0.05) [14]. It is a simplified calculation of anthropogenic Gd input with a known natural geological Gd concentration. The quantified equations [34] are as follows:

Gd_SN_/Gd*_SN_ = Gd_SN_/(0.4Nd_SN_ + 0.6Dy_SN_) (1)

Gd* = Gd*_SN_ × Gd_PAAS_ (2)

Gd_anthr._ = Gd − Gd^*^ (3)

where ‘SN’, ‘*’ and ‘anthr.’ denote the PAAS-normalized REEs concentrations, natural geological background concentrations, and anthropogenic contributed Gd concentrations. Additionally, the monthly data were tested for normal distribution. The average value was used for calculation if the data obeyed normal distribution. Otherwise, the median value was used.

## 3. Results and Discussion

### 3.1. The Distribution of Dissolved REEs

The concentrations of total dissolved REEs (ΣREE) in three streams are listed in Table 1. For the Youyu stream, the mean ΣREE concentration was 31.22 ng/L with a minimum value of 22.02 ng/L and a maximum value of 48.93 ng/L. The ΣREE concentrations of the Baiyan stream ranged from 26.91 to 46.58 ng/L, while the Jinzhong stream presented a rather wide range of the ΣREE, from 30.19 to 145.53 ng/L. With the high- concentration of Gd in Jinzhong, the ΣREE were less than 150 ng/L. The natural source of dissolved REEs in rivers is the rock weathering [35]. Consequently, dissolved REEs leached from the labile REE-bearing minerals inherit the REE characteristics from the rocks [36]. However, due to the coprecipitation and complexation, the signatures of dissolved REEs are different from those of rocks. Dissolved REEs form complexes in rivers that are sensitive to major ions and pH. Under high pH and cation concentration conditions, colloids in rivers decrease, resulting in the less REE complexes in water [37,38]. Therefore, the ΣREE of the three streams are lower than those of other rivers, such as Amazon, Indus, and Mississippi [39].

**Figure 2:**
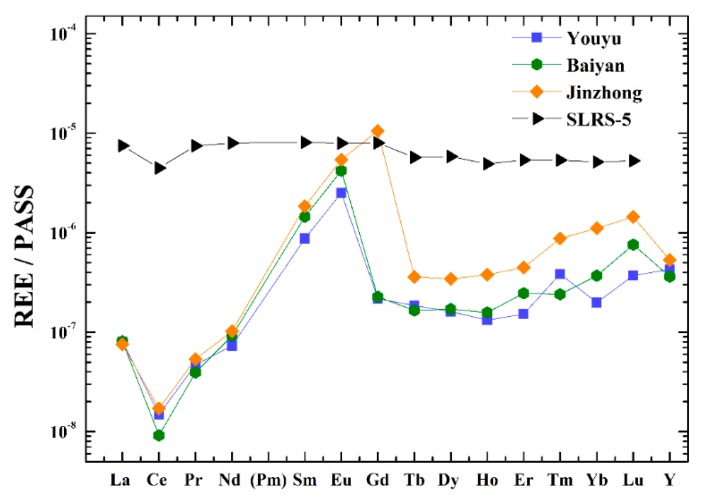
REE patterns of the three streams (Youyu, Baiyan, and Jinzhong) and SLRS-5. All streams show Ce anomalies, Eu anomalies and Sm anomalies.

Figure 2 illustrates the patterns of REEs for three streams. Comparing the PAAS-normalized REE pattern of SLRS-5, it was clear that the REE patterns in the three streams were not smooth with negative and positive anomalies for certain REEs. Usually, HREE preferentially form complexes in solution [38,40] and are less readily removed from the water. In comparison to the LREE, HREE complexes are weakly reactive to the adsorption reaction [14]. Therefore, all the samples showed enrichment of HREE.

### 3.2. The REE Anomaly

#### 3.2.1. The Negative Ce Anomaly

Ce and Eu anomalies are commonly produced during weathering and transport processes in the fractionation of REEs [7,41]. The negative Ce anomalies could be explained by, not only weathering, but also other factors, such as pH, redox, and organic ligand complexing [42,43,44]. When trivalent Ce transform into tetravalent Ce, the negative Ce anomalies occur, and then, the tetravalent Ce form of CeO_2_ is preferentially removed in rivers [45]. However, under acidic conditions, CeO_2_ readily reacts with H^+^ to form Ce ions again. The reaction is as follows [38,42]: CeO_2_ + 4H^+^ +e^−^ = Ce^3+^ + 2H_2_O. The Ce anomalies could be affected by pH. Xu and Han [24] reported that the negative Ce anomalies in the Xijiang River occurred with high pH. In addition, in the Wujiang River, high- pH and alkaline rivers, also showed negative Ce anomalies [38]. In particular, three streams showed the negative Ce anomalies that was identical for the rivers in carbonate regions (Figure 2), such as the Xijiang River [24], Wujiang River [38], and Lake Aha [3]. Thus, the negative Ce anomalies in three streams not only are sensitive to oxidative conditions, but also depend on the pH of the rivers in carbonate regions. 

#### 3.2.2. The Positive Eu Anomaly

All the streams samples exhibited positive Eu anomalies. Positive Eu anomalies are considered to be dependent on lithology [18,46]. Furthermore, Eu^2+^ is preferentially released into solution during the weathering of host lithologies [24,47]. Consistent with the Xijiang River and the Corrente River, the lithology of the study areas showed the positive Eu anomalies in three streams. The positive Eu anomalies in the three streams may also have been due to an anthropogenic source. Itoh et al. [15] reported the positive anomalies of Eu in the Sakai River, Japan and the source of Eu was not found (a “potential” anthropogenic source). However, there was no obvious source of Eu in the study areas. 

#### 3.2.3. The Positive Sm Anomaly

At present, Sm is widely applied in the manufacturing of luminescent materials and permanent magnets. Sm is also of great importance in the ceramics industry [48,49,50]. Sm generally exists in the form of colloids and nanoparticles in water [33,51,52,53]. Both natural processes, weathering, and anthropogenic input cause the positive Sm anomalies in waters. Studies in the Seine illustrated that Sm anomalies was derived from the product effluent of cracking catalysis, such as La [52], and the anomalies of anthropogenic Sm was also observed in the Han River [33]. However, Sm anomalies were found in two main tributaries of the Garonne River and anomalies were only observed at the two sampling points for several months [51]. All the streams in the studied areas were characterized with positive Sm anomalies, but the corresponding point source (e.g., fluid catalytic converters) were not found. Due to the consistency of the background and the same trends in the Sm anomalies of the three streams (Figure 2), the Sm anomalies need further research to determine the corresponding source.

#### 3.2.4. The Positive Gd Anomaly

**Figure 3:**
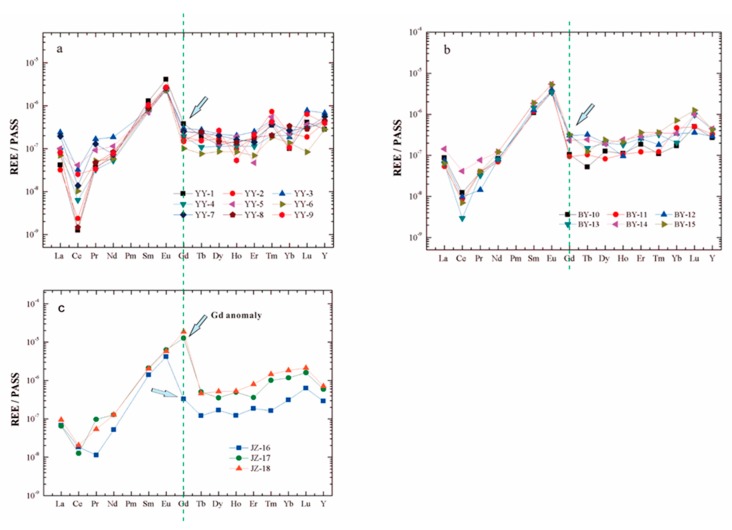
The PASS shale-normalized rare earth elements (REE) patterns in Youyu (**a**), Baiyan (**b**), and Jinzhong (**c**), and the Gd of JZ-17 and JZ-18 are the main sampling points affected by human factors.

As shown in Figure 2, a distinct positive Gd anomaly was only found in Jinzhong, while there was no clear anomaly in Youyu and Baiyan. Table 1 lists the Gd concentrations of the samples. The Gd concentrations of study areas increased from 0.47 to 1.77 ng/L, 0.49 to 1.50 ng/L, and 1.54 to 86.65 ng/L for Youyu, Baiyan, and Jinzhong, respectively. Compared to that in Youyu and Baiyan, Gd in Jinzhong presented a rather wide range (85.11 ng/L). The Gd concentration in the Jinzhong accounted for up to 60% of all REEs, while it accounted for only 3% of all REEs in the other streams. The concentrations of Gd in the Jinzhong were much higher than those reported in other rivers flowing through carbonate rocks in Guizhou Province. The Gd concentrations of Lapinghe and Zhangjiang in Guiyang accounted for 3.11% and 2.78% of all REEs, respectively [24]. In the middle reaches of the Wujiang River located in the Guiyang area, the Gd values accounted for 3.5% of the REEs [38]. Wang et al. showed that the concentration of Gd was 13.9% of all REEs in Lake Aha 6 years ago [3]. The natural REE fraction seemed unlikely to produce significant positive Gd anomalies. In addition, the shale-normalized REE patterns showed enriched Gd in the Jinzhong, while no distinct positive Gd was observed in the other two streams (Figure 2 and Figure 3a,b). This result indicated that Jinzhong had important anthropogenic Gd inputs.

The positive Gd anomalies are considered to be present in densely populated, industrial, and medically developed areas [11,15,17,54,55]. As previously noted, the Youyu and Baiyan are surrounded by villages. There are no MRI instruments near both streams. As a typical urban small stream, the Jinzhong flows through an area with a population of approximately 60,000 and whit a hospital, and wastewater treatment plants (WWTPs). 

In general, the ratio of Gd_SN_/Gd*_SN_ is used to predict Gd anomalies. A Gd_SN_/Gd*_SN_ > 1 indicates a positive Gd anomaly. In contrast, a Gd_SN_/Gd*_SN_ < 1 suggests a negative anomaly [17,21,29]. Equations (1) was used to quantify all water samples to determine the Gd anomalies in this research. As shown in Table 1, there were smooth variations in the Gd anomaly in Youyu and Baiyan, from 0.78 to 3.86 and from 0.96 to 2.05, with mean values of 1.78, and 1.56, respectively. However, notably, the maximum Gd_SN_/Gd*_SN_ ratios in Jinzhong were observed in the three streams. Characterized by significant positive Gd anomalies, the Gd_SN_/Gd*_SN_ ratios for the Jinzhong ranged from 2.75 to 51.87, approximately 20 fold and 23 fold higher than that of the Youyu, and Baiyan, respectively. Even higher than that of the Alzette River’s upstream (mean ratio, 4.84) and downstream (mean ratio, 6.57), and these anomalies were impacted by industrial, waste incineration, hospital sewage, etc.

### 3.3. Anthropogenic Gd in Jinzhong

During the past few decades, rivers, lakes, and WWTPs have been documented as having distinct positive Gd anomalies [13,14,15,17,22,51,56]. The anomaly was first revealed by Bau and Dulski in 1996 [17], derived from the gadopentetic acid (Gd-DTPA) used in magnetic resonance imaging (MRI) in the hospitals. The Han River, that flows through the Seoul Capital Area with over 25 million people, had anthropogenic Gd (Gd_anthr._) 30–70% [33]. Surrounded by a megalopolis, southern San Francisco Bay also reported anthropogenic Gd [14]. The calculation of Gd_anthr._ concentrations can be simplified with a known, natural, geological Gd concentration. Through Equations (2) and (3), Gd_anthr._ values are presented for each sampling point in Table 1. The extremely high Gd_anthr._ proportions occurred in Jinzhong at 63.64% (JZ-16), 97.94% (JZ-17) and 98.07% (JZ-18). The proportions of anthropogenic Gd even exceed that of the Rhine River (Gd_anthr._, 28–91%) [52], San Francisco South Bay (Gd_anthr._ > 70%) [14], and Han River (Gd_anthr._, 30–84%) [33]. 

When an MRI examination is performed, a patient of that weighs approximately 70 kg needs an injection of 15 mL of Gd, and 80% of the Gd is excreted rapidly after the examination [22,51]. Then the complexation of Gd enters the rivers through the effluent of WWTPs after excretion from the human body. Gd complexation has high water solubility and stability. Its conservation makes it possible to exist for several weeks or even months in an aquatic environment [14,17,21,51]. The Jinyang Wastewater Treatment Plant (JWTP), with a capacity of 50,000 m^3^/d is near Jinzhong. The proposal related to the treatment of environment in Jinzhong showed that approximately 146,000 m^3^/d of sewage emissions were released into the stream [57]. It is important that nearly 100,000 m^3^/d of sewage is directly discharged into the Jinzhong with a relatively limited sewage treatment capacity [57,58]. In addition, the sewer network is damaged and more than 50% of the sections have been destroyed because of the structures resulting in an increase in the pollutants, such as Gd, in Jinzhong [57]. Accumulating from hospitals and domestic sewage, the Gd in Jinzhong showed significant Gd anomalies.

**Figure 4:**
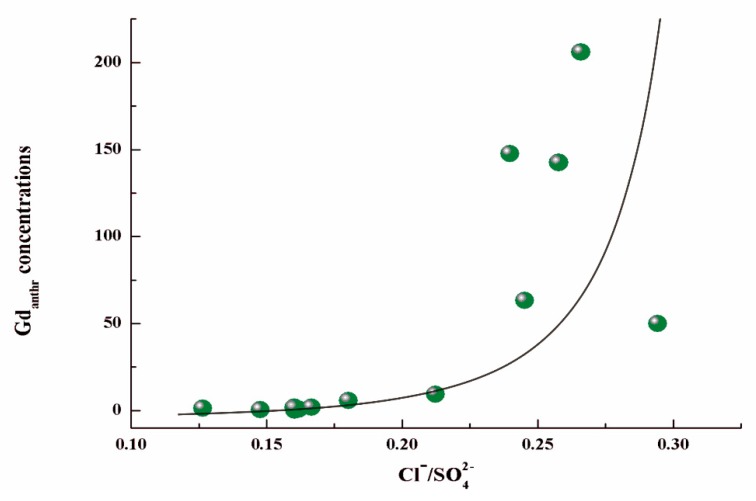
Relationship between Gd_anthr._ concentrations and Cl^−^/SO_4_^2−^ in Jinzhong.

The positive correlation between Gd_anthr._ and Cl^−^/SO_4_^2−^ (r = 0.9017, *p* < 0.05) is shown in Figure 4. SO_4_^2−^, and HCO_3_^−^ were the main anions in the study areas (carbonate regions) [59]. To eliminate the water chemistry effect between the anions, the normalized Cl^-^ by SO_4_^2−^ represented the Cl^−^ concentrations [60]. The Gd_anthr._ concentration increased with the Cl^−^ concentrations. The Gd_anthr._ in Jinzhong could be explained as a form of Gd complexation with Cl^−^, which was consistent with the results of the study by Song in 2017 [33].

Gd concentrations displayed a gradual increase from upstream to downstream in the Jinzhong stream. Distinct spatial disparities were observed with increasing Gd_SN_/Gd*_SN_ ratios from upstream to downstream (Table 1). The upstream samples displayed slightly more positive Gd anomalies than those at the other sampling points. In the vicinity of Guanshanhu Park, an ecological wetland, the upstream sampling point was surrounded by enriched woodland. With few hospitals and and a smaller population, the Gd_SN_/Gd*_SN_ ratio upstream was only 2.75, which was lower than those at the other points in Jinzhong. The estimated anthropogenic contributions of Gd ranged from 0.98 ng/L to 84.98 ng/L in Jinzhong. From upstream to downstream, Gd_anthr._ concentrations were elevated to 84 ng/L. Notably, a certain amount of anthropogenic Gd input was found between JZ-17 and JZ-18, resulting in a positive Gd anomaly. To locate the actual site of the anthropogenic Gd anomaly, the area flowing through the JZ-17 and JZ-18 was observed in more detail. Between JZ-17 and JZ-18, the Guiyang Third People’s Hospital with a magnetic resonance imaging (MRI) may explain the anthropogenic input in Jinzhong. MRI is usually used to identify early tumor and lymph nodes by adding a paramagnetic contrast agent, Gd-DTPA [61,62]. The Gd-containing medical wastewater enters the public swage system after treatment at wastewater plants [22,33,51]. 

**Figure 5:**
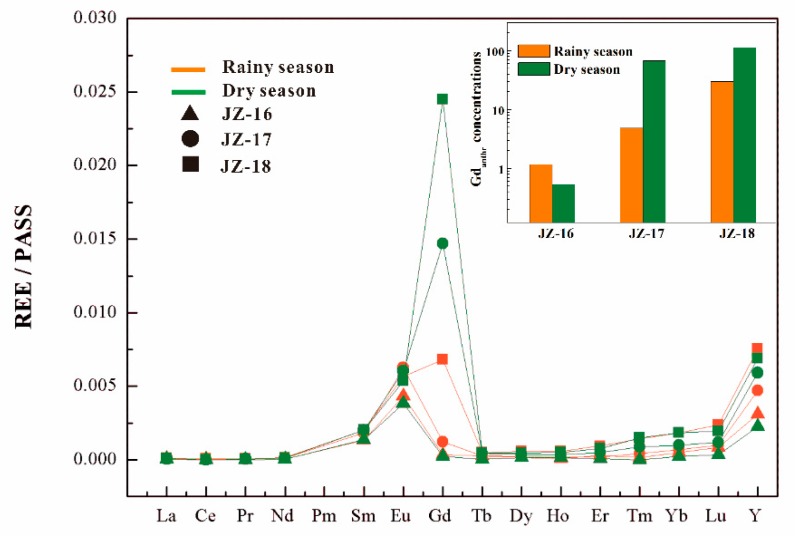
Significant seasonal variations in Gd in Jinzhong.

Figure 5 illustrates significant seasonal variations in dissolved Gd in Jinzhong. For the downstream WWTP (JZ-17 and JZ-18), the rainy season samples showed lower Gd concentrations and Gd_anthr._ than those of the dry season. In contrast, upstream of Jinzhong (JZ-16), the Gd concentrations and Gd_anthr._ were higher in the rainy season than in the dry season. This interesting observation could be explained as follows: Downstream, the anthropogenic REEs were mainly derived from the WWTP point source. In the rainy seasons, the anthropogenic REEs were diluted by rainwater. However, upstream, the anthropogenic REEs mainly came from the nonpoint sources. In the dry seasons, there was less input from run-off containing anthropogenic REEs from nonpoint sources to upstream water, while in rainy seasons, this contribution could be larger, producing the higher REE concentrations observed upstream (JZ-16). 

## 4. Conclusions

Our pilot study of three streams in Guiyang, a medium-sized city in southwestern China, demonstrates HREE enrichment, due to the strong adsorption reaction of LREE. The negative Ce anomalies, and the positive Sm and Eu anomalies are observed in the three streams. The cause of the Ce anomaly was due to, not only weathering, but also pH in alkaline rivers. The positive Eu anomalies were considered dependent on lithology. 

The PASS-normalized REE patterns of Jinzhong demonstrated the presence of anthropogenic Gd in the stream (Jinzhong) flowing through the densely populated area. Ranging from 0.98 to 84.98 ng/L, the anthropogenic Gd which is derived from gadopentetic acid (Gd-DTPA) used in MRI, was attributed to sewage discharge from municipalities, and hospitals. Anthropogenic Gd as a micropollutant can be traced back to the Guiyang Third People’s Hospital, a point source, with magnetic resonance imaging in the radiology department. Because of its high stability and low particulate reactivity, Gd offers a low-cost tracer for predicting medical wastewater and derived substances.

As the MRI market in China is rapidly expanding and expected to grow even further, the discharge of anthropogenic Gd into the river system will increase in the future decades. While, the biological effects of REEs are still poorly understood, it is important to perform further studies on the possible health effects, bioavailability, and ecotoxicology of anthropogenic Gd.

## Figures and Tables

**Table 1:** The rare earth elements (REE) concentrations (ng/L) in the three streams (Youyu, Baiyan and Jinzhong).

Sample ^1^	pH	Cl^-^	SO_4_^2−^	La	Ce	Pr	Nd	Sm	Eu	Gd	Tb	Dy	Ho	Er	Tm	Yb	Lu	Y	ΣREE	Gd/Gd *^,2^	Gd_anthr._^3^
Youyu		**mg/L**	**mg/L**																		
YY-1	7.74	6.57	226.29	1.58	0.10	0.00	2.14	7.17	4.41	1.77	0.15	0.57	0.15	0.48	0.14	0.28	0.18	7.61	26.72	3.86	1.31
YY-2	8.14	4.50	333.76	1.22	0.19	0.41	2.34	4.43	2.64	0.87	0.12	0.80	0.10	0.53	0.29	0.63	0.08	10.96	25.63	1.43	0.26
YY-3	8.16	4.80	420.67	9.06	2.57	1.47	6.24	4.34	2.52	1.38	0.21	1.02	0.20	0.71	0.19	0.52	0.33	18.18	48.93	1.45	0.43
YY-4	7.79	4.42	328.93	3.49	0.50	0.28	1.76	3.97	2.51	0.89	0.08	0.53	0.11	0.32	0.08	0.58	0.13	10.58	25.82	2.15	0.48
YY-5	7.98	3.87	176.59	3.84	3.29	0.81	3.87	3.88	2.72	1.14	0.18	0.59	0.19	0.13	0.22	0.63	0.16	10.91	32.57	2.02	0.58
YY-6	7.98	5.22	111.31	2.66	0.81	0.46	1.94	4.39	2.47	0.47	0.06	0.40	0.08	0.20	0.07	0.39	0.04	7.59	22.02	1.36	0.13
YY-7	7.36	6.66	89.53	7.44	1.09	1.14	2.47	4.82	2.78	1.17	0.18	0.94	0.16	0.40	0.15	0.75	0.13	15.09	38.71	1.69	0.48
YY-8	7.63	4.58	303.33	2.99	0.12	0.41	2.81	4.87	2.86	0.71	0.19	0.68	0.14	0.52	0.08	0.95	0.12	12.06	29.51	1.27	0.15
YY-9	7.25	6.11	213.77	3.10	2.01	0.30	2.63	5.77	3.19	0.69	0.12	1.23	0.05	0.61	0.17	0.30	0.28	10.59	31.03	0.78	0.00
Baiyan																					
BY-10	8.13	7.27	267.95	3.32	0.97	0.35	2.81	6.00	3.75	0.49	0.04	0.59	0.11	0.53	0.04	0.48	0.22	7.22	26.91	0.96	0.00
BY-11	8.22	7.30	86.76	2.06	0.68	0.35	2.35	6.46	4.03	0.43	0.08	0.38	0.10	0.35	0.05	1.31	0.22	8.80	27.66	1.21	0.08
BY-12	8.13	8.01	106.31	2.82	0.77	0.13	2.71	6.95	4.21	1.42	0.25	0.91	0.10	0.73	0.07	0.95	0.15	7.77	29.93	2.05	0.73
BY-13	7.88	9.84	122.69	2.39	0.23	0.28	2.65	8.06	3.69	1.41	0.12	0.92	0.18	0.75	0.13	0.57	0.42	11.29	33.10	2.02	0.71
BY-14	7.75	13.03	100.72	5.50	3.29	0.68	4.08	10.28	5.88	1.09	0.19	0.89	0.24	0.82	0.14	0.96	0.42	12.11	46.58	1.44	0.33
BY-15	8.22	15.17	116.15	2.46	0.56	0.35	4.10	10.50	5.71	1.50	0.10	1.11	0.21	1.03	0.15	1.98	0.54	11.36	41.65	1.69	0.61
Jinzhong																					
JZ-16	7.76	10.97	94.52	2.56	1.47	0.10	1.76	7.69	4.50	1.54	0.09	0.78	0.12	0.52	0.07	0.88	0.27	7.83	30.19	2.75	0.98
JZ-17	8.02	13.74	67.22	2.49	1.00	0.85	4.30	11.73	6.78	59.30	0.39	1.64	0.49	1.02	0.41	3.29	0.69	16.02	110.41	48.74	58.08
JZ-18	8.31	23.38	94.36	3.59	1.61	0.47	4.31	11.28	6.24	86.65	0.35	2.40	0.51	2.27	0.59	5.18	0.91	19.16	145.53	51.87	84.98
SLRS-5 ^4^				232.07	280.95	52.92	214.58	37.88	7.93	31.88	3.98	22.71	4.07	12.33	1.61	10.05	1.63				

^1^ YY: Youyu Stream, BY: Baiyan Stream, JZ: Jinzhong Stream. ^2^ Gd_SN_/Gd*_SN_ = Gd_SN_/(0.4Nd_SN_ + 0.6Dy_SN_) (see Section 2.3.). ^3^ Gd_anthr._ = Gd − Gd^*^(see Section 2.3.). ^4^ natural water samples from the National Research Council of Canada (unpublished data).
